# Adapting an immune competent mouse model for glioblastoma T cell therapy

**DOI:** 10.1186/2051-1426-2-S3-P35

**Published:** 2014-11-06

**Authors:** Tania Rodriguez-Cruz, Zhongzhen Yi, Stephen Gottschalk, Simone Krebs

**Affiliations:** 1 Baylor College of Medicine/Texas Childrens Hospital/ Center for Cell and Gene Therapy, Houston, TX, United States

## 

Glioblastoma (GBM) is the second most common, but most aggressive, primary brain tumor. Despite aggressive multimodal therapy consisting of surgery, radiation, and chemotherapy, the outcome of patients with GBM remains poor with 5-year survival rates of <10%. Therefore, new, targeted treatments are needed to improve outcomes, and adoptive T cell immunotherapy has the potential to fulfill this need. T cells genetically modified with chimeric antigen receptors (CARs) specific for cell surface antigens expressed in GBM such as HER2, EphA2, EGFRvIII, and IL13R (a1 and a2) have shown promising anti-GBM activity in preclinical GBM models. However, the majority of studies have been conducted in xenograft models that do not recapitulate the immunosuppressive tumor microenvironment. The goal of this project is now to adapt the established GL261 immune competent murine GBM model for the preclinical optimization of GBM-targeted CAR T cell therapies.

Since GL261 were readily killed by IL13R-specific human CAR T cells, we generated a retroviral vector encoding an IL13R-specific murine CAR with a CD28.CD3z endodomain. CD3-activated splenocytes were transduced with VSV-G pseudotyped retroviral particles, sorted, and used for functional *in vitro *studies. In contrast to non-transduced T cells, IL13R-specific murine CAR T cells recognize GL261, as judged by their ability to proliferate, secrete cytokines and kill GL261 cells in co-culture assays. IL13R-negative cells did not activate IL13R-specific murine CAR T cells, confirming antigen specificity. Animal experiments are in progress to evaluate the safety and efficacy of IL13R-specific murine CAR T cells *in vivo*.

In summary, our results indicate that is feasible to develop an immune competent murine GBM-targeted T cell immunotherapy model. Such a model will not only facilitate the translation of these therapies into the clinic, but will also allow us to realistically study how to best combine T cell therapy with other GBM-targeted therapies.

